# 755. *Clostridioides difficile* Testing and the Use of Laxatives in Immunocompromised Adults

**DOI:** 10.1093/ofid/ofab466.952

**Published:** 2021-12-04

**Authors:** Amanda Al-Bahou, Rod Quilitz, Yanina Pasikhova, John Greene

**Affiliations:** 1 Moffitt Cancer Center, Tampa, Florida; 2 Moffitt Cancer Center and Research Institute, Tampa, Florida

## Abstract

**Background:**

*Clostridioides difficile* infection (CDI) rates have plateaued at historical highs in the United States since 2010 and remains a major health problem. While optimal CDI testing remains unclear, current literature recommends testing patients whose symptoms are not clinically attributable to underlying conditions, e.g., laxatives. At Moffitt Cancer Center, a soft-stop alert was implemented to alert the provider if the patient received a laxative within the previous 48 hours of CDI testing. We aim to evaluate the incidence of CDI rates with prior laxative use in immunocompromised patients, as well as, the impact of the soft-stop alert in reducing CDI testing.

**Methods:**

Retrospective, single-center, review of adult patients who were tested for CDI after the implementation of the soft-stop alert from October 1, 2020 to December 21, 2020. These patients were compared to a historical cohort of patients who were tested for CDI prior to the alert implementation from October 1, 2019 to December 31, 2019. The primary outcome was the percent of patients that received a laxative within 48 hours of CDI testing pre-alert compared to post-alert. Secondary outcomes included the percent of colonization versus active infection in this immunocompromised population, number and type of laxatives administered prior to testing, and the frequency of alert and reduction of CDI tests ordered. A cost-benefit analysis was also performed.

**Results:**

In the historical cohort (n=480), 14.8% received a laxative within 48 hours of CDI testing (Figure 1). Within patients who received a laxative in this group, 4.2% had a definitive active infection. After the alert was implemented, a total of 630 CDI tests were ordered from October 1, 2020 to December 31, 2020, and the alert was fired for 123 (19.5%) tests ordered (Figure 2). Of the tests where the alert was fired, the CDI test was removed for 42.3% and continued for 57.7% of orders resulting in savings of &3,263. In this cohort, 5.6% of patients had a definitive active CDI infection who received a laxative and testing was continued (Figure 3).

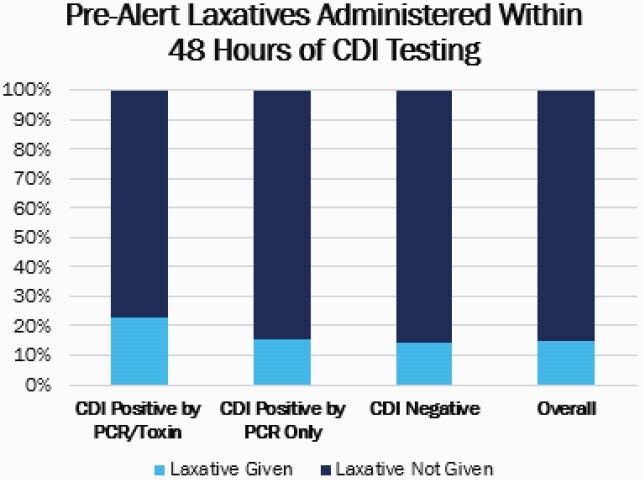

Figure 2: CDI Test and Laxative Administration Alert

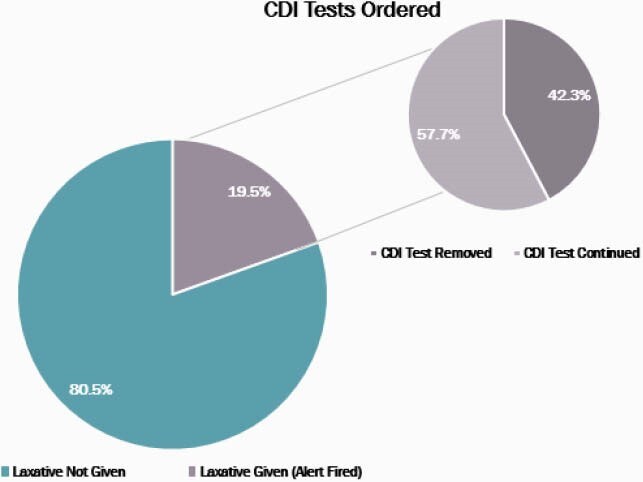

Figure 3: Post-Alert Laxatives Administered and CDI Test Result

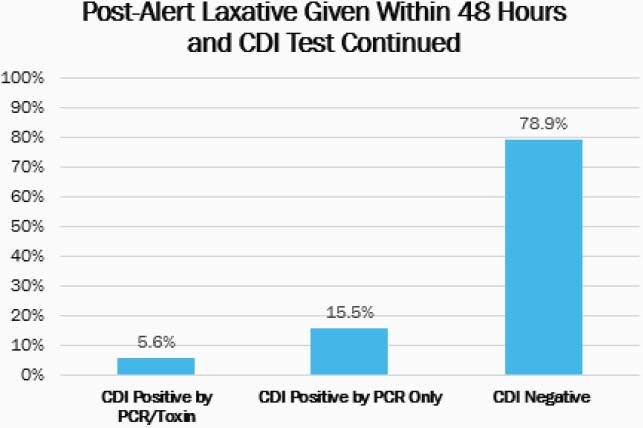

**Conclusion:**

The soft-stop alert implemented reduced CDI testing in patients who received a laxative in the last 48 hours correlating with a financial benefit, improvement in guideline adherence, and reduction in laboratory labor.

**Disclosures:**

**All Authors**: No reported disclosures

